# Synthesis of Fe_2_O_3_ Nanorod and NiFe_2_O_4_ Nanoparticle Composites on Expired Cotton Fiber Cloth for Enhanced Hydrogen Evolution Reaction

**DOI:** 10.3390/molecules29133082

**Published:** 2024-06-28

**Authors:** Sun Hua, Sayyar Ali Shah, Noor Ullah, Nabi Ullah, Aihua Yuan

**Affiliations:** 1 School of Environmental & Chemical Engineering, Jiangsu University of Science and Technology, Zhenjiang 212003, China; 2 Department of Inorganic and Analytical Chemistry, Faculty of Chemistry, University of Lodz, Tamka 12, 91-403 Lodz, Poland; nabi.ullah@chemia.uni.lodz.pl

**Keywords:** hydrogen evolution reaction, Fe_2_O_3_-NiFe_2_O_4_, electrocatalyst, synergistic effect, heterointerface

## Abstract

The design of cheap, noble-metal-free, and efficient electrocatalysts for an enhanced hydrogen evolution reaction (HER) to produce hydrogen gas as an energy source from water splitting is an ideal approach. Herein, we report the synthesis of Fe_2_O_3_ nanorods–NiFe_2_O_4_ nanoparticles on cotton fiber cloth (Fe_2_O_3_-NiFe_2_O_4_/CF) at a low temperature as an efficient electrocatalyst for HERs. Among the as-prepared samples, the optimal Fe_2_O_3_-NiFe_2_O_4_/CF-3 electrocatalyst exhibits good HER performance with an overpotential of 127 mV at a current density of 10 mA cm^−2^, small Tafel slope of 44.9 mV dec^−1^, and good stability in 1 M KOH alkaline solution. The synergistic effect between Fe_2_O_3_ nanorods and NiFe_2_O_4_ nanoparticles of the heterojunction composite at the heterointerface is mainly responsible for improved HER performance. The CF is an effective substrate for the growth of the Fe_2_O_3_-NiFe_2_O_4_ nanocomposite and provides conductive channels for the active materials’ HER process.

## 1. Introduction

The use of fossil fuels for energy requirements not only declines its reserves but also causes environmental problems [1,2]. The developments of green and renewable energy sources are alternative strategies to deal with these issues [3,4]. Hydrogen is regarded as an efficient and promising energy source due to its high mass-specific energy density, environmental friendliness, and zero-carbon emission on combustion [5]. Hydrogen produced from water splitting is one of the most suitable and sustainable processes [6,7]. However, the electrochemical water splitting hydrogen evolution reaction (HER) proceeds at the cathode and requires some overpotential. A highly active and stable catalyst is required to reduce the overpotential for the HER [8,9,10]. Pt-based materials are highly active electrocatalysts and only require small overpotentials for HERs [11,12]. However, the high cost of precious metals and the scarcity of reserves are obstacles to large-scale applications. It is essential to develop cheaper, readily available, and efficient electrocatalyst materials for HERs [13,14,15].

Over the past few decades, many efficient precious-metal-free electrocatalytic materials have been designed and investigated for HERs with good performance and stability [16,17,18]. The transition 3d metals, especially Fe, Co, and Ni-based materials, have attracted the attention of researchers because of their low price and excellent catalytic performance [19,20,21]. For example, Mondal et al. reported a NiO electrocatalyst for HER performance and good stability in alkaline solution. However, single 3d metal-based materials require large overpotentials as compared to bimetallic-based composites. The 3d transition metal nanocomposites were reported as having enhanced electrocatalytic performance due to the synergetic effect at the interface of heterogeneous composites [22,23]. For example, Shi et al. reported the NiFe oxides-based materials as bifunctional electrocatalysts for both HERs and OERs in an alkaline medium. However, most FeNi-based materials possess low electroconductivity and still do not reach the benchmark.

To further enhance the HER performance, the heterojunction catalytic components can be grown on the conductive substrate. Nickel foam or carbon materials such as carbon cloth, carbon fiber paper, carbon nanotubes, or graphene are used as substrates for the growth of NiFe catalyst. For example, Yan et al. [24]. synthesized a layered porous Ni_x_Fe-S/NiFe_2_O_4_ heterogeneous electrocatalyst on a three-dimensional carbon cloth by electrodeposition. The optimal sample of Ni_1/5_Fe-S/NiFe_2_O_4_ showed the best HER performance due to the synergistic effect of the bimetallic heterostructure. Our group reported Ni_3_S_2_/Fe_2_O_3_/NC on nickel foam through a facile one-step thermal process as bifunctional catalysts for enhanced HERs and OERs. Therefore, heterojunction catalysts grown on substrates become outstanding electrodes for electrocatalytic HER performance [25,26,27]. However, these substrates are relatively expensive. It is highly desirable to use cost-effective substrates for the growth of catalytic nanomaterials. The NiFe-based composites grown on cotton fiber (CF) cloth are rarely reported in electrolysis systems, especially in HERs.

Here, we report the synthesis of a Fe_2_O_3_ nanorod/NiFe_2_O_4_ nanoparticle composite on the cotton fiber cloth of expired shirts (Fe_2_O_3_-NiFe_2_O_4_/CF) for enhanced HER performance. The composite consists of NiFe_2_O_4_ nanoparticles loaded on Fe_2_O_3_ nanorods to form a heterointerface catalyst. The optimized Fe_2_O_3_-NiFe_2_O_4_/CF catalyst shows excellent HER performance with an overpotential of only 127 mV at a current density of 10 mA cm^−2^, a small Tafel slope of only 44.9 mV dec^−1^, and good stability in 1M KOH solution. Its excellent performance is attributed to the synergistic effect between Fe_2_O_3_ nanorods and NiFe_2_O_4_ nanoparticles and the electron transfer between Ni and Fe species at the interface in composites, resulting in high active sites produced for HER performance.

## 2. Results and Discussions

### 2.1. Synthesis and Characterization

The Fe_2_O_3_-NiFe_2_O_4_/CF composite was prepared by a simple one-step process. During synthesis, the (NiCl_2_∙6H_2_O) and (FeCl_2_∙4H_2_O) precursors were dissolved into Fe^2+^ and Ni^2+^ ion aqueous mediums. Fe^2+^ reacted with oxygen dissolved in the water to grow Fe_2_O_3_ nanorods on the CF. Simultaneously, Ni^2+^ and some Fe^2+^ also reacted with oxygen and formed NiFe_2_O_4_ nanoparticles. Most of these NiFe_2_O_4_ nanoparticles were loaded on Fe_2_O_3_ nanorods. Finally, the Fe_2_O_3_-NiFe_2_O_4_/CF heterointerface composite material was formed on the CF. The amounts of different catalysts deposited on the CF during synthesis are listed in Table S2.

**Figure 1:**
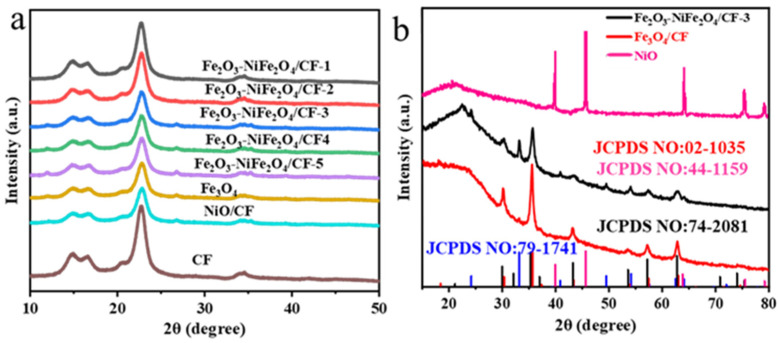
(**a**) XRD pattern of as-prepared samples and CF. (**b**) XRD pattern of Fe_2_O_3_-NiFe_2_O_4_/CF-3, Fe_3_O_4_/CF, and NiO/CF samples’ powder removed from the surface of the CF.

The crystal structure of the prepared samples was characterized and analyzed using the XRD technique (Figure 1a). The XRD patterns of samples showed peaks concentrated at 22.65°, 14.9°, 16.6°, and 34.4° indexed to (200), (1–10), (110), and (004) of crystalline cellulose, respectively. Due to the high diffraction peaks of the CF substrate, the diffraction peaks of the other materials cannot be observed. The nanomaterial of the Fe_2_O_3_-NiFe_2_O_4_/CF-3 sample was collected from the surface of the substrate by sonication, and the XRD pattern was recorded (Figure 1b). The peaks around 24.1°, 33.1°, 40.8°, 49.4°, 54.1°, 62.4.0°, and 64.0° corresponded to the (012), (104), (113), (024), (116), (214), and (300) crystal planes of Fe_2_O_3_ (JCPDS NO.79-1741), respectively. The diffraction peaks at approximately 30.2°, 35.6°, 43.3°, 53.8°, 57.3°, and 63.0° are consistent with the (220), (311), (400), (422), (511), and (440) crystal planes of NiFe_2_O_4_ (JCPDS NO.74-2081). These diffraction peaks showed that the material is a composite of Fe_2_O_3_ and NiFe_2_O_4_. However, when Fe salts are used alone in the reaction mixture, the XRD patterns of the collected powder are matched with JCPDS NO.02-1035 of cubic Fe_3_O_4_, while when using Ni precursors in the reaction mixture, the XRD patterns are consistent with JCPDS NO.44-1159 of hexagonal NiO. All the samples showed broad peaks at 2θ around 24°. This may be due to amorphous carbon materials formed at 150 °C on CF and peeling from the CF surface during sonication.

**Figure 2:**
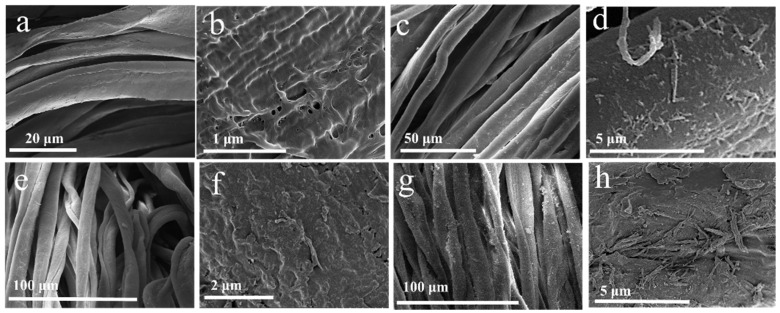
SEM image of (**a**,**b**) CF, (**c**,**d**) Fe_3_O_4_/CF, (**e**,**f**) NiO/CF, and (**g**,**h**) Fe_2_O_3_-NiFe_2_O_4_/CF-3 samples at different magnifications.

The morphology and microstructure of the samples were observed by SEM. The pure CF surface was cleaned, and no nanomaterial was observed on it (Figure 2a,b). Dipping the CF in FeCl_2_ solution, its surface was modified, and nanorods could be observed on the surface (Figure 2c,d). On the other hand, only nanoparticles could be seen on the CF (Figure 2e,f) when it was immersed in the NiCl_2_ solution. When aqueous solutions of both FeCl_2_ and NiCl_2_ were used for the synthesis, the nanoparticles were observed on the nanorods’ surface (Figure 2g,h). Some nanoparticles were also observed in the surface on the CF. The morphology of the Fe_2_O_3_-NiFe_2_O_4_/CF-1 (Figure S1a), Fe_2_O_3_-NiFe_2_O_4_/CF-2 (Figure S1b), Fe_2_O_3_-NiFe_2_O_4_/CF-4 (Figure S1c), and Fe_2_O_3_-NiFe_2_O_4_/CF-5 (Figure S1d) samples was very similar when using different concentrations of FeCl_2_. However, it is obvious that the amount of nanorods gradually increased on the CF with the increasing concentration of FeCl_2_ in the reaction mixture. From the SEM observation of catalysts, it is suggested that the nanorods and nanoparticles are Fe_2_O_3_ and NiFe_2_O_4_, respectively. The atomic contents of Ni and Fe in the composite samples were confirmed by ICP-OES. The atomic ratio of Ni and Fe is 1:0.17, 1:0.48,1:0.83, 1:1.13, and 1:1.47 in Fe_2_O_3_-NiFe_2_O_4_/CF-1, Fe_2_O_3_-NiFe_2_O_4_/CF-2, Fe_2_O_3_-NiFe_2_O_4_/CF-3, Fe_2_O_3_-NiFe_2_O_4_/CF-4, and Fe_2_O_3_-NiFe_2_O_4_/CF-5, respectively. The slightly high atomic contents of Ni compared to the final composition of the Fe_2_O_3_-NiFe_2_O_4_ catalyst may be due the presence of Ni material contamination.

**Figure 3:**
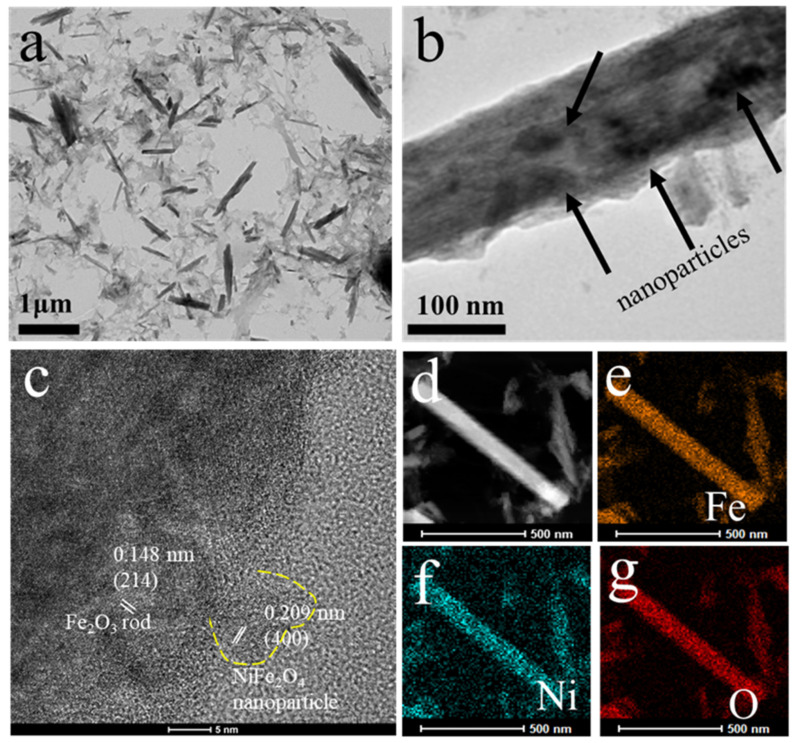
(**a**,**b**) TEM and (**c**) HRTEM images of Fe_2_O_3_-NiFe_2_O_4_/CF-3 sample. (**d**) Dark-field TEM image and element mapping. (**e**) Fe, (**f**) Ni, and (**g**) O images of Fe_2_O_3_-NiFe_2_O_4_/CF-3 sample.

The Fe_2_O_3_-NiFe_2_O_4_ sample removed from the surface of the CF was characterized by TEM to further confirm morphology. As shown in Figure 3a, the nanorods are distributed in a light gray material-like structure. The diameters of the nanorods are tens of nanometers and the length is a few micrometers. The light-gray-like structure may be amorphous carbon materials and peeled from the CF during sonication. The nanoparticles loaded on the nanorods can be seen in Figure 3b. The HRTEM images showed that the lattice fringes of nanoparticles are 0.209 nm, corresponding to the (400) lattice plane of NiFe_2_O_4_ (Figure 3c). The lattice fringes with a spacing of 0.148 nm are consistent with the (214) lattice plane of Fe_2_O_3_ nanorods. The HRTEM results further indicate that the composites consisted of Fe_2_O_3_ nanorods and NiFe_2_O_4_ nanoparticles. Figure 3d displays the dark-field TEM images and corresponding elemental mapping images of Fe (Figure 3e), Ni (Figure 3f), and O (Figure 3g). It can be seen that the Fe and O signals are uniformly observed in the nanorods, and the Ni signal is uniformly observed on the nanorods due to the very small size of the nanoparticles, which further confirms that the composite material is composed of Fe_2_O_3_ nanorods and NiFe_2_O_4_ nanoparticles.

**Figure 4:**
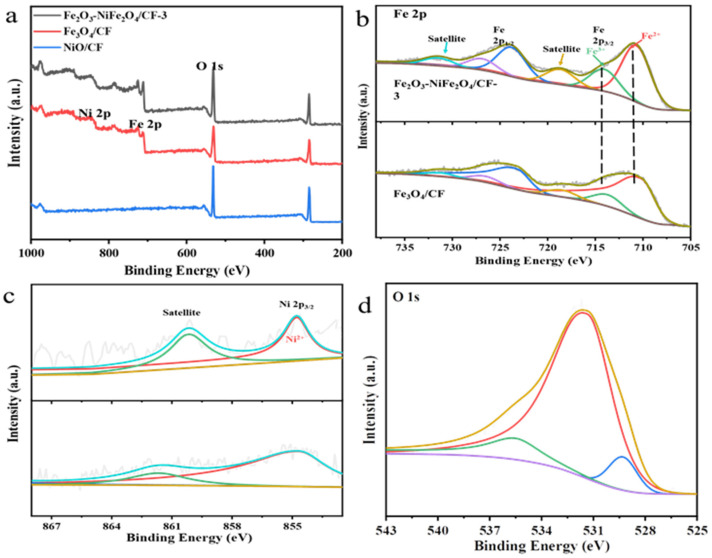
(**a**) Survey XPS spectrum of Fe_2_O_3_-NiFe_2_O_4_/CF-3, Fe_3_O_4_/CF, and NiO/CF samples. (**b**) High-resolution XPS spectra of Fe 2p region of Fe_2_O_3_-NiFe_2_O_4_/CF-3 and Fe_3_O_4_/CF catalysts. (**c**) High-resolution XPS spectra of Ni 2p region of Fe_2_O_3_-NiFe_2_O_4_/CF-3 and NiO/CF catalysts and (**d**) O 1s spectrum of Fe_2_O_3_-NiFe_2_O_4_/CF-3 product.

XPS analysis of the sample was performed to determine its valence state and elemental composition. The survey XPS spectrum of the Fe_2_O_3_-NiFe_2_O_4_/CF-3 samples showed peaks of the Fe, Ni, C, and O elements, compared with the survey XPS spectrum of Fe_3_O_4_/CF and NiO/CF (Figure 4a). The high-resolution XPS spectrum in the Fe 2p region of the Fe_2_O_3_-NiFe_2_O_4_/CF-3 samples can be deconvoluted into different peaks (Figure 4b). The binding energy peaks at 710.9 and 714.2 eV of the Fe 2p_3/2_ band suggested the existence of Fe^2+^ and Fe^3+^ oxidation states, respectively [28,29]. The binding energy peaks 723.9 and 727.1 eV were also associated with oxidation states of Fe^2+^ and Fe^3+^ of the Fe 2p_1/2_ band, respectively [30]. The peaks at 718.8 and 727.1 eV can be assigned to satellites of Fe 2p [31]. The high-resolution XPS spectrum in the Fe 2p region of Fe_3_O_4_/CF showed binding energy peaks at 714.1 and 710.8 eV for the Fe 2p_3/2_ and 726.9 and 723.7 eV for the Fe 2p_1/2_ band. The Fe 2p_3/2_ and Fe 2p_1/2_ bands of Fe_3_O_4_/CF also existed in the Fe^2+^ and Fe^3+^ oxidation states. The satellites peaks of Fe 2p show peaks at binding energies of 718.7 and 727 eV. The binding energy of the Fe 2p_3/2_ and Fe 2p_1/2_ bands slightly shifted towards the lower energy of Fe_3_O_4_/CF compared to Fe_2_O_3_-NiFe_2_O_4_/CF-3. This indicates that there is interaction and electron transfer between Ni and Fe species in composites. The XPS spectra of the Ni 2p region of Fe_2_O_3_-NiFe_2_O_4_/CF-3 and NiO is shown in Figure 4c. The peak centered on 854.7 eV is related to Ni 2p_3/2_ of the Ni^2+^ valence states [32]. The peaks at a binding energy of 860.2 eV were the satellite of Ni 2p. There was no obvious change in the Ni 2p spectra of the Fe_2_O_3_-NiFe_2_O_4_/CF-3 and NiO samples. As shown in Figure 4d, the O 1s spectrum can be attributed to O_2_^2−^ at 529.4 eV, representing the M-O bond. The peak of 531.9 eV may be due to the absorption of oxygen or water molecules, and 534.9 eV indicates the O-O bond [33,34,35].

### 2.2. Electrocatalytic HER Performance

**Figure 5:**
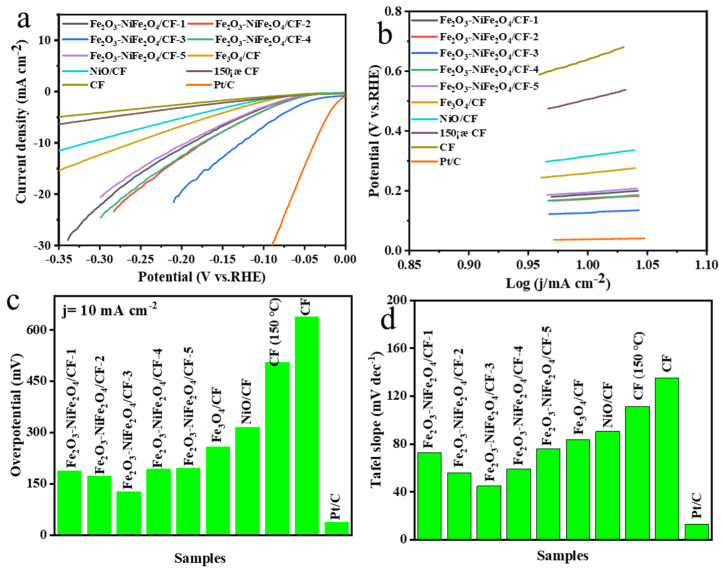
(**a**) LSV curves of as-prepared samples, Pt/C, CF, and CF heated at 150 °C (CF 150 °C). (**b**) Comparison of overpotentials at a current density of 10 mA cm^2^ of different samples. (**c**) The Tafel slopes and (**d**) comparison of Tafel slopes of different samples.

The HER performance of as-prepared samples and Pt/C catalyst was studied in 1.0 M KOH solution using a three-electrode setup. The LSV curves of the catalysts are shown in Figure 5a. The commercial Pt/C catalyst has the best HER performance of all the tested samples, with an overpotential of 38 mV at a current density of 10 mA cm^−2^. Among the as-prepared samples, Fe_2_O_3_-NiFe_2_O_4_/CF-3 displayed good HER performance and needed an overpotential of 127 mV to reach a current density of 10 mA cm^−2^ (Figure 5b). The Fe_2_O_3_-NiFe_2_O_4_/CF-3 catalyst exhibited comparable or better performance in alkaline solutions than the previously reported NiFe_2_O_4_ catalysts (Table S3). The Fe_2_O_3_-NiFe_2_O_4_/CF-1 and Fe_2_O_3_-NiFe_2_O_4_/CF-2 composites showed low HER performance with overpotentials of 188 mV and 173 mV at a current density of 10 mA cm^−2^, respectively. On the other hand, Fe_2_O_3_-NiFe_2_O_4_/CF-4 and Fe_2_O_3_-NiFe_2_O_4_/CF-5 required overpotentials of 176 and 195 mV at a current density of 10 mA cm^−2^, respectively. The high HER performance of the Fe_2_O_3_-NiFe_2_O_4_/CF-3 catalyst in composite samples is due to the optimal amount of catalyst loaded on the CF after 3 h of the reaction, and it has relatively high numbers of the heterointerface between Fe_2_O_3_ nanorods and NiFe_2_O_4_ nanoparticles as compared to other composite catalysts. However, the HER performance of composites is much better than using the Fe or Ni salt precursors alone in the reaction mixture, and Fe_3_O_4_/CF and NiO displayed overpotentials of 259 and 316 mV at a current density of 10 mA cm^−2^, respectively. The CF without any metal materials loaded showed very poor HER performance. These results suggest that enhanced HER performance of Fe_2_O_3_-NiFe_2_O_4_ composites is attributed to the synergistic effect between heterointerface Fe_2_O_3_ nanorods and NiFe_2_O_4_ nanoparticles.

The Tafel slope value indicates the study of the reaction kinetics and the mechanism of the catalysts. Tafel slopes are derived from the Tafel plots by fitting linear portions of the curves (Figure 5a) with the Tafel equation (η = a + blog*j*), where η, j, b, and a are the overpotential, current density, Tafel slope, and constant, respectively). The Tafel slope of the Fe_2_O_3_-NiFe_2_O_4_/CF-3 composite was 44.9 mV dec^−1^ (Figure 5d) and much lower than Fe_2_O_3_-NiFe_2_O_4_/CF-1 (72.6 mV dec^−1^), Fe_2_O_3_-NiFe_2_O_4_/CF-2 (55.8 mV dec^−1^), Fe_2_O_3_-NiFe_2_O_4_/CF-4 (59.3 mV dec^−1^), Fe_2_O_3_-NiFe_2_O_4_/CF-5 (75.8 mV dec^−1^), Fe_3_O_4_/CF (83.7 mV dec^−1^), NiO/CF (90.5 mV dec^−1^), CF (150 °C) (111.3 mV dec^−1^), and CF (135.3 mV dec^−1^) except Pt/C (32.8 mV dec^−1^). This indicates that the Fe_2_O_3_-NiFe_2_O_4_/CF-3 composite has faster reaction kinetics in the as-prepared sample and therefore exhibited better HER properties.

A small Tafel slope is conducive to practical application since it will lead to a faster increment of the HER rate with low overpotential. According to classical theory and recent reports, in alkaline/neutral conditions, the Volmer and Heyrovsky (Equations (1) and (2)) reactions show Tafel slope values of 120 and 40 mV dec^−1^, respectively. In comparison, the Tafel slope value of the Tafel reaction is about 30 mV dec^−1^ and remains the same for all pH values [36,37].

H_2_O + e^−^ + M → M-H_ads_ + OH^−^ (Volmer) (1)

H_2_O + e^−^ + M-H_ads_ → H_2_ + OH^−^ + M (Heyrovsky) (2)

M-H_ads_ + M-H_ads_ →H_2_ + M (Tafel) (3)

where M is a catalytic active material and H_ads_ represents an adsorbed hydrogen on the surface of the electrocatalyst. The molecular hydrogen produces either combinations of Volmer–Heyrovsky reactions or Volmer–Tafel reactions in the HER process. According to the Tafel slope values, the Tafel slope value of our Fe_2_O_3_-NiFe_2_O_4_/CF-3 composite was 44.9 mV dec^−1^ and possibly follows the Volmer–Heyrovsky reaction pathway for the HER process and rate determination step [38,39]. The other Fe_2_O_3_-NiFe_2_O_4_/CF composites’ values were from 55.8.2 to 83.7 mV dec^−1^ and indicated that the composites follow the same reaction pathway during the HER process.

**Figure 6:**
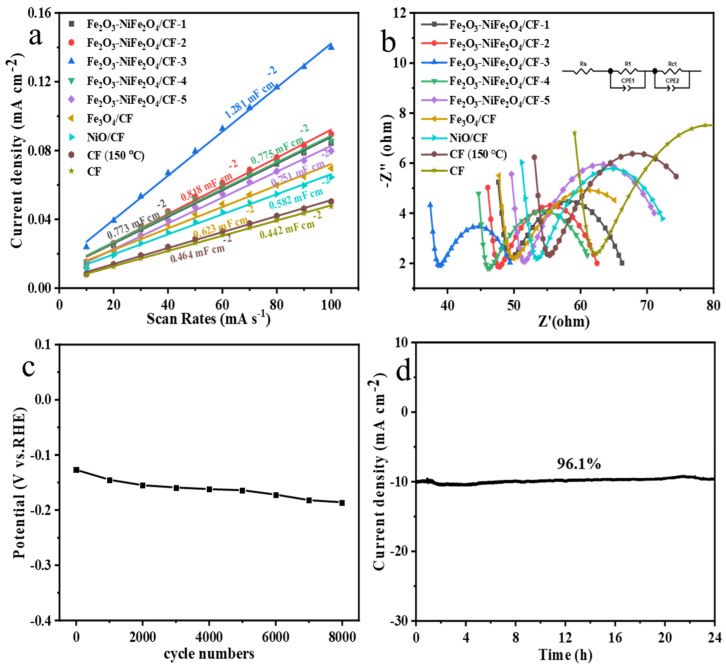
(**a**) Cdl values of the different samples. (**b**) Nyquist plot of samples and inset equivalent circuit fitting experimental data. (**c**) Overpotential (V vs. RHE) at constant current density of 10 mA cm^−2^ of Fe_2_O_3_-NiFe_2_O_4_/CF-3 sample before and after 1000–8000 CV cycles and (**d**) i–t test of Fe_2_O_3_-NiFe_2_O_4_/CF-3 composite.

Furthermore, the ECAS of these samples was assessed by electrochemical double-layer capacitors (*C_dl_*). The C_dl_ values are positively correlated with ECAS and are therefore commonly used to describe ECAS. Figure S2a–i showed CV curves of different samples with different scan rates and C*_dl_* values were calculated according to these CV curves. The C*_dl_* value (Figure 6b) decreased in the order of Fe_2_O_3_-NiFe_2_O_4_/CF-3 (1.281 mF cm^−2^) > Fe_2_O_3_-NiFe_2_O_4_/CF-2 (0.818 mF cm^−2^) > Fe_2_O_3_-NiFe_2_O_4_/CF-4 (0.775 mF cm^−2^) > Fe_2_O_3_-NiFe_2_O_4_/CF-1 (0.773 mF cm^−2^) > Fe_2_O_3_-NiFe_2_O_4_/CF-5 (0.751 mF cm^−2^). (Figure 6a) > Fe_3_O_4_/CF (0.623 mF cm^−2^), NiO/CF (0.582mF cm^−2^) > CF (0.464 mF cm^−2^) > CF (150 °C) (0.442 mF cm^−2^). The Fe_2_O_3_-NiFe_2_O_4_/CF-3 sample has the highest C*_dl_* value and this indicates a high number of active sites for enhanced HER performance.

Electrochemical impedance spectroscopy (EIS) of the prepared sample was measured in order to determine electron transfer at the electrode/electrolyte interface, as shown in Figure 6b. The Nyquist plots were fitted (illustrated inset Figure 6b) in the equivalent circuit. The charge transfer resistance (R_ct_) of the prepared samples was revealed. The charge transfer resistance (R_ct_) values of Fe_2_O_3_-NiFe_2_O_4_/CF-1, Fe_2_O_3_-NiFe_2_O_4_/CF-2, Fe_2_O_3_-NiFe_2_O_4_/CF-3, Fe_2_O_3_-NiFe_2_O_4_/CF-4, Fe_2_O_3_-NiFe_2_O_4_/CF-5, Fe_3_O_4_/CF, NiO/CF, CF, and CF (150 °C) were 20.69, 17.8, 13.91, 19.11, 25.73, 25.98, 24.41, 29.18, and 37.43 Ω, respectively. Among all samples, the Fe_2_O_3_-NiFe_2_O_4_/CF-3 sample exhibited lower R_ct_ values than other materials and this indicates more efficient charge transfer at the electrode/electrolyte interface for improved catalytic performance.

The stability of the electrocatalyst is an important criterion for the HER. The stability of the Fe_2_O_3_-NiFe_2_O_4_/CF-3 composite catalyst was confirmed by CV cycle and amperometry (i-t) tests. The LSV curves initially and after 1000–8000 CV cycles were measured, as shown in Figure S3. There is a slight reduction in overpotential at a current density of 10 mV cm^−2^ as the number of cycles increased (Figure 6c). This suggests that Fe_2_O_3_-NiFe_2_O_4_/CF-3 exhibited good stability under alkaline conditions. The nanorods can be observed on surface of the CF in the SEM images after 8000 CV cycles (Figure S4a). This indicates that the morphology of the nanorods is not obviously changed. The TEM image confirmed that nanoparticles were on the surface of nanorods (Figure S4b). The i–t test was measured with 24 h of continuous operation (Figure 6d). The i-t test showed that 96.1% of the current density was maintained after 24 h. This further confirmed the good stability of the sample.

## 3. Experimental Section

### 3.1. Materials and Chemical Reagents

Nickel chloride hexahydrate (NiCl_2_∙6H_2_O) and potassium hydroxide (KOH) were purchased from McLean Biotechnology Limited, Shanghai, China. Ferrous chloride tetrahydrate (FeCl_2_∙4H_2_O) and anhydrous ethanol were purchased from Sinopharm Chemical Reagent Co., Ltd., Shanghai, China. Deionized water was used in all experimental work. An expired cotton shirt of Uniqlo brand was used.

### 3.2. Preparation of Fe_2_O_3_-NiFe_2_O_4_/CF Composites

CF was cut into pieces of 1 × 3 cm^2^ from a 100% cotton expired commercial T-shirt. The CF pieces were cleaned by washing with a deionized water/ethanol mixture under sonication for 20 min and then dried at 150 °C for 12 h in a nitrogen atmosphere in order to enhance its conductivity. An amount of 0.5 g of FeCl_2_∙4H_2_O and 0.6 g of NiCl_2_∙6H_2_O were dissolved in 150 mL deionized water and dried, and the CF was suspended in it. The solution was heated at 40 °C under magnetic stirring in an oil bath. After 2 h, the CF was removed, cleaned several times with deionized water and ethanol, and then placed in a vacuum drying oven at 60 °C for 12 h, to obtain the Fe_2_O_3_-NiFe_2_O_4_/CF catalyst. By changing the addition amounts of FeCl_2_∙4H_2_O salt, the influence on the catalytic performance was explored. The catalyst was named Fe_2_O_3_-NiFe_2_O_4_/CF-X (where X indicates the weight of FeCl_2_∙4H_2_O in grams, Table S1).

### 3.3. Synthesis of the Fe_3_O_4_/CF and NiO/CF

The Fe_3_O_4_/CF was synthesized with the same procedure and only FeCl_2_∙4H_2_O was dissolved in deionized water, while for the NiO/CF sample, NiCl_2_∙6H_2_O was used as the initial precursor in the reaction mixture.

### 3.4. Material Characterization

The morphology and microstructure of the samples were observed by scanning electron microscopy (SEM, XL30-FEG, Tokyo, Japan) and transmission electron microscopy (TEM, JEM-2010, Massachusetts, United State of America). The crystal phase composition of the samples was studied by an X-ray diffractometer (XRD, Bruker D-8 Advanced diffractometer, Tokyo, Japan). The elemental composition and valence states of the samples were studied by X-ray photoelectron spectroscopy (XPS, AXIS ULTRA, Suzhou, China). The Fe and Ni ion contents in the samples were determined by an inductively coupled plasma optical emission spectrometer (ICP-OES, Vista-MPX, Massachusetts, United State of America).

### 3.5. Electrocatalytic Hydrogen Evolution Performance

Electrochemical tests were performed at room temperature in 1.0 M KOH solution using an electrochemical workstation (CHI 760E, Shanghai Chenhua Co., Ltd. Shanghai, China) of a three-electrode system. A graphite electrode, Hg/HgO electrode, and CF-based samples were used as the counter, reference, and working electrode, respectively. The catalyst was activated by 50 cyclic voltammetry (CV) scans at a scanning rate of 100 mV s^−1^ between 0 and −1.5 V, followed by linear sweep voltammetry (LSV) at a scanning rate of 5 mV s^−1^. Stability tests were performed after every 1000–8000 CV cycles at a scan rate of 100 mV S^−1^, and then LSV was measured after every 1000 CV cycles again under the same conditions. The electrochemical active surface area (ECSA) of the samples was determined from double-layer capacitance C_dl_ values under potential windows of 0~0.1 V. Electrochemical impedance spectroscopy (EIS) measurements were performed in the frequency range of 100 kHZ to 100 mHZ. All measured potentials were calibrated to the reversible hydrogen electrode (RHE) according to the following formula: E(_RHE)_ = E_(Hg/HgO)_ + 0.098 + 0.059 pH (1.0 M KOH).

## 4. Conclusions

In summary, we have successfully prepared Fe_2_O_3_-NiFe_2_O_4_/CF composites at a low temperature for an efficient HER electrocatalyst. The optimal Fe_2_O_3_-NiFe_2_O_4_/CF-3 electrocatalyst displayed enhanced HER performance with an overpotential of 127 mV at a current density of 10 mA cm^−2^ and small Tafel slope of 44.9 mV dec^−1^. The Fe_2_O_3_-NiFe_2_O_4_/CF sample also showed good stability and durability in alkaline solutions. We believe that the enhanced HER performance is due to the synergistic effect between Fe_2_O_3_ nanorods and NiFe_2_O_4_ nanoparticles at the heterointerface. The CF can be an effective substrate for the growth of the Fe_2_O_3_-NiFe_2_O_4_ nanocomposite and provide conductive channels for the active materials’ HER process.

## Data Availability

The original contributions presented in the study are included in the article and Supplementary Materials. Further inquiries can be directed to the corresponding authors.

## Supplementary Materials

The following supporting information can be downloaded at: https://www.mdpi.com/article/10.3390/molecules29133082/s1. Table S1. Initial precursor used to synthesize different Fe_2_O_3_-NiFe_2_O_4_/CF-X catalysts. Table S2. Catalytic deposited on the CF during synthesis. Figure S1. SEM image of (a) Fe_2_O_3_-NiFe_2_O_4_/CF-1, (b) Fe_2_O_3_-NiFe_2_O_4_/CF-2, (c) Fe_2_O_3_-NiFe_2_O_4_/CF-4, (d), Fe_2_O_3_-NiFe_2_O_4_/CF-5 samples. Table S3. Catalytic performance comparison of Fe_2_O_3_-NiFe_2_O_4_/CF-3 sample with previously reported catalysts. Figure S2. (a–i) CV curves of as-synthesized samples, CF and CF (150 °C) between −0.1 and 0 V vs. Hg/HgO at different scan rate mV s −1 in 1.0 M alkaline solution. (g) The electrochemical surface area of catalysts was determined from Cdl. Cdl values was calculated by plotting the ΔJ = (Ja-Jc) at 0.05 V vs. Hg/HgO against various scan rates, the 2Cdl is equal to the slope. Figure S3. LSV curves of Fe_2_O_3_-NiFe_2_O_4_/CF-3 catalyst before and after different CV cycles. Figure S4. (a) SEM and (b) TEM images of Fe_2_O_3_-NiFe_2_O_4_/CF-3 sample after 8000 CV cycles. References [39,40,41,42,43,44,45,46] are cited in the supplementary materials.
