# Supplementary material for: Synthesis of Fe2O3 Nanorod and NiFe2O4 Nanoparticle Composites on Expired Cotton Fiber Cloth for Enhanced Hydrogen Evolution Reaction
Source: Molecules. 2024 Jun 28;29(13):3082. doi: 10.3390/molecules29133082 (PMC11243087; doi:10.3390/molecules29133082)
Supplement: Supplementary file 1 [file molecules-29-03082-s001.zip › molecules-3028994-supplementary.pdf]

Synthesis of Fe_2O_3 Nanorod and $NiFe_2O_4$ Nanoparticle Composites on Expired Cotton Fiber Cloth for Enhanced Hydrogen Evolution Reaction

Sun Hua [1], Sayyar Ali Shah [1,*], Noor Ullah [1], Nabi Ullah [2] and Aihua Yuan [1,*]

[1] School of Environmental & Chemical Engineering, Jiangsu University of Science and Technology,
Zhenjiang 212003, China; 18261970601@163.com (S.H.); noorullahmian57@gmail.com (N.U.)

[2] Department of Inorganic and Analytical Chemistry, Faculty of Chemistry, University of Lodz, Tamka 12, 91-403 Lodz, Poland; nabi.ullah@chemia.uni.lodz.pl

* Correspondence: sayyarali83@just.edu.cn (S.A.S.), aihua.yuan@just.edu.cn (A.Y.)

Table S1. Initial precursor used to synthesize different Fe_2O_3-$NiFe_2O_4$/CF-X catalysts.

Sample	$FeCl_2·4H_2O$(g)	$NiCl_2·6H_2O$(g)
Fe_2O_3-$NiFe_2O_4$/CF-1	0.1	0.6
Fe_2O_3-$NiFe_2O_4$/CF-2	0.3	0.6
Fe_2O_3-$NiFe_2O_4$/CF-3	0.5	0.6
Fe_2O_3-$NiFe_2O_4$/CF-4	0.7	0.6
Fe_2O_3-$NiFe_2O_4$/CF-5	0.9	0.6
Fe_3O_4/CF	0.5	—
NiO/CF	—	0.6

Table S2. Catalytic deposited on the CF during synthesis.

Sample	Catalysts deposits on CF (mg)
Fe_2O_3-$NiFe_2O_4$/CF-1	2.1
Fe_2O_3-$NiFe_2O_4$/CF-2	3.2
Fe_2O_3-$NiFe_2O_4$/CF-3	4.5
Fe_2O_3-$NiFe_2O_4$/CF-4	5.8
Fe_2O_3-$NiFe_2O_4$/CF-5	6.3
Fe_3O_4/CF	1.6
NiO/CF	1.5

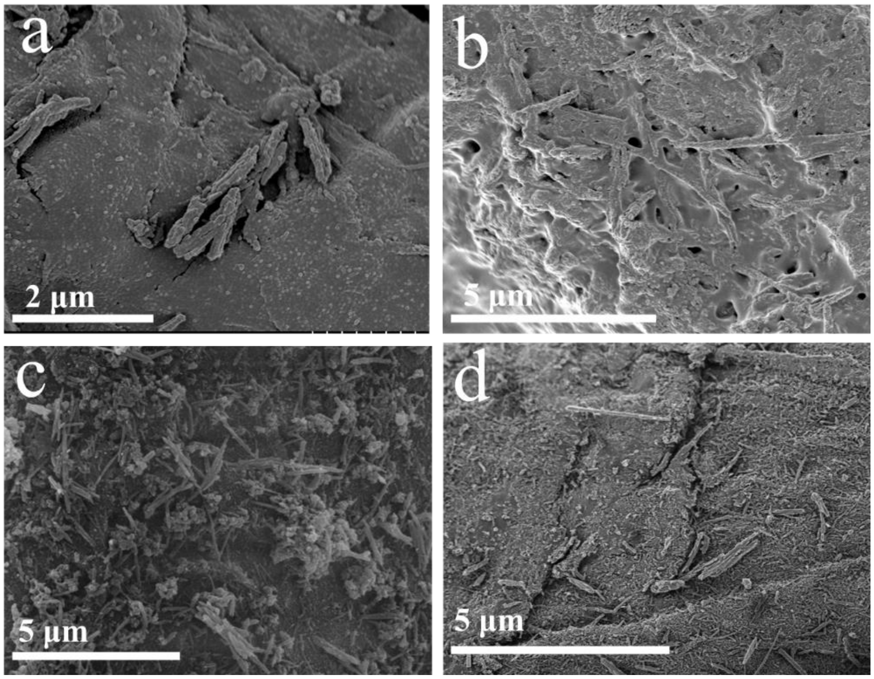

Figure S1. SEM image of (a) Fe_2O_3-$NiFe_2O_4$/CF-1, (b) Fe_2O_3-$NiFe_2O_4$/CF-2, (c) Fe_2O_3-$NiFe_2O_4$/CF-4, (d), Fe_2O_3-$NiFe_2O_4$/CF-5 samples.

Table S3. Catalytic performance comparison of Fe_2O_3-$NiFe_2O_4$/CF-3 sample with previously reported catalysts.

Catalysts	η_{10} (mV)	Tafel slope (mV dec^{-1})	Reference
Fe_2O_3-$NiFe_2O_4$/CF-3	127	44.9	**This work**
$NiFe_2O_4$/Ti_3C_2	173	112.2	1
$NiFe_2O_4$/CB	187	85.8	2
$NiFe_2O_4$/CoNi-S	149	49.8	3
$NiFe_2O_4$@N-rGO-CC	188	218.5	4
$NiFe_2O_4$ @N/rGO-800	157	49.7	5
$NiFe_2O_4$@MOF-74	403	106	6
$Co_{0.5}Ni_{0.5}Fe_2O_4$	174	91	7
$NiFe_2O_4$/NF	135	122.7	8

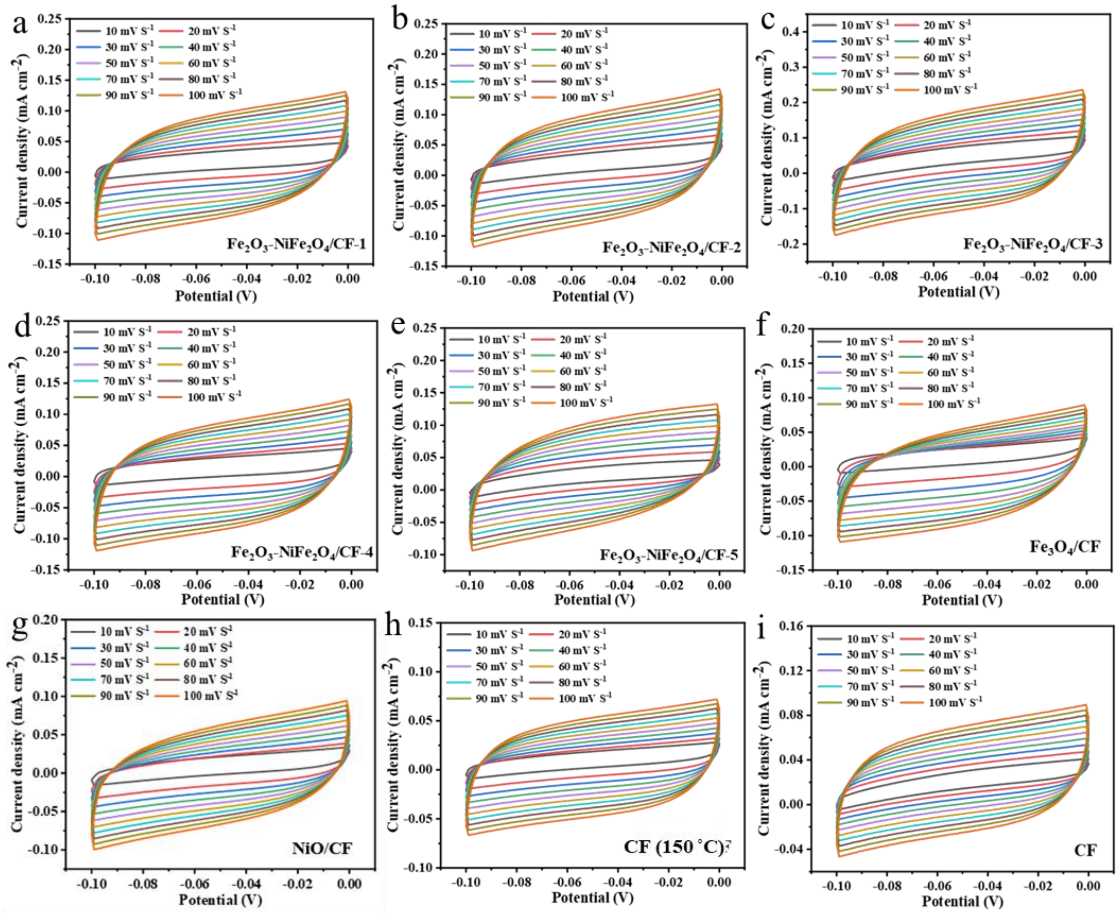

Figure S2. (a-i) CV curves of as-synthesized samples, CF and CF (150 ˚C) between -0.1 and 0 V *vs*. Hg/HgO at different scan rate mV s^{-1} in 1.0 M alkaline solution. (g) The electrochemical surface area of catalysts was determined from C_{dl}. C_{dl} values was calculated by plotting the $\Delta J = (J_a - J_c)$ at 0.05 V *vs*. Hg/HgO against various scan rates, the $2C_{dl}$ is equal to the slope.

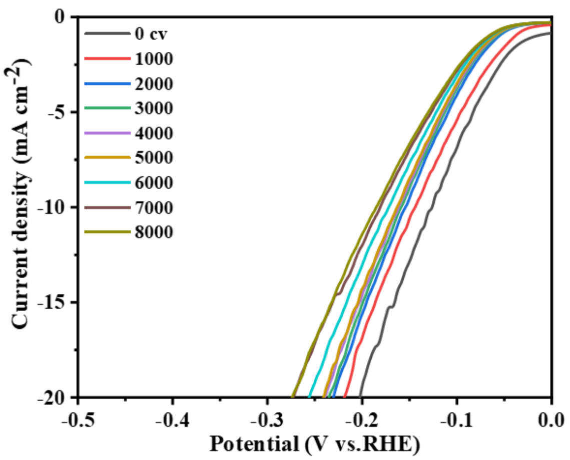

Figure S3. LSV curves of Fe_2O_3-$NiFe_2O_4$/CF-3 catalyst before and after different CV cycles.

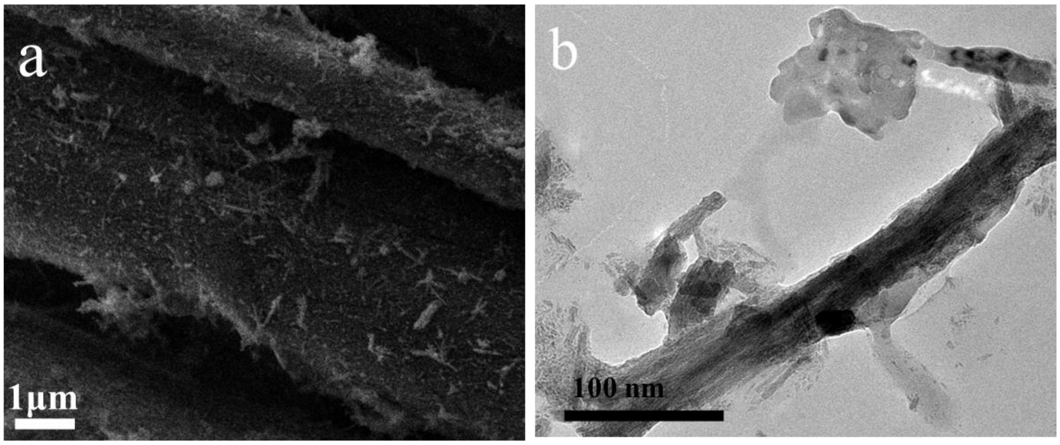

Figure S4. (a)SEM and (b) TEM images of Fe_2O_3-$NiFe_2O_4$/CF-3 sample after 8000 CV cycles

References

1. Shinde, P. V.; Mane, P.; Chakraborty, B.; Rout, C. S. J. J. o. C.; Science, I., Spinel $NiFe_2O_4$ nanoparticles decorated 2D Ti3C2 MXene sheets for efficient water splitting: Experiments and theories. *Journal of Colloid and Interface Science* **2021,** *602*, 232-241.
2. Munonde, T. S.; Zheng, H.; Matseke, M. S.; Nomngongo, P. N.; Wang, Y.; Tsiakaras, P. J. R. E., A green approach for enhancing the electrocatalytic activity and stability of $NiFe_2O_4$/CB nanospheres towards hydrogen production. *Renewable Energy* **2020,** *154*, 704-714.
3. Shi, Y.; Feng, X.; Guan, H.; Zhang, J.; Hu, Z. J. I. J. o. H. E., Porous sunflower plate-like $NiFe_2O_4$/CoNi–S heterostructure as efficient electrocatalyst for overall water splitting. *International Journal of Hydrogen Energy* **2021,** *46* (12), 8557-8566.
4. Zhang, K.; Jiang, P.; Gu, Q.; Leng, Y.; Zhang, P. J. E.; Fuels, Design of a High-Efficiency Bifunctional Electrocatalyst: Rich-Nitrogen-Doped Reduced Graphene Oxide-Modified Carbon Cloth-Growing Nickel–Iron Complex Oxides for Overall Water Splitting. *Energy & Fuels* **2022,** *36* (9), 4911-4923.
5. Cao, L.; Li, Z.; Su, K.; Zhang, M.; Cheng, B. J. J. o. e. c., Rational design of hollow oxygen deficiency-enriched NiFe2O4@ N/rGO as bifunctional electrocatalysts for overall water splitting. *Journal of energy chemistry* **2021,** *54*, 595-603.
6. Zheng, H.-b.; Chen, H.-h.; Wang, Y.-l.; Gao, P.-z.; Liu, X.-p.; Rebrov, E. V. J. A. A. M.; Interfaces, Fabrication of magnetic superstructure $NiFe_2O_4$@ MOF-74 and its derivative for electrocatalytic hydrogen evolution with AC magnetic field. *ACS Applied Materials & Interfaces* **2020,** *12* (41), 45987-45996.
7. Sadiq Mohamed, M. J.; Caliskan, S.; Gondal, M. A.; Almessiere, M. A.; Baykal, A.; Slimani, Y.; Elsayed, K. A.; Hassan, M.; Auwal, I. A.; Khan, A. Z. J. A. A. N. M., Se-doped magnetic Co–Ni spinel ferrite nanoparticles as electrochemical catalysts for hydrogen evolution. *ACS Applied Nano Materials* **2023,** *6* (9), 7330-7341.
8. Du, X.; Tan, M.; Wei, T.; Kobayashi, H.; Song, J.; Peng, Z.; Zhu, H.; Jin, Z.; Li, R.; Liu, W. J. C. E. J., Highly efficient and robust nickel-iron bifunctional catalyst coupling selective methanol oxidation and freshwater/seawater hydrogen evolution via CO-free pathway. *Chemical Engineering Journal* **2023,** *452*, 139404.
